# CRISPR/Cas9-Mediated Gene Therapy for Glioblastoma: A Scoping Review

**DOI:** 10.3390/biomedicines12010238

**Published:** 2024-01-21

**Authors:** Emir Begagić, Hakija Bečulić, Nermin Đuzić, Amina Džidić-Krivić, Ragib Pugonja, Asja Muharemović, Belma Jaganjac, Naida Salković, Haso Sefo, Mirza Pojskić

**Affiliations:** 1 Department of General Medicine, School of Medicine, University of Zenica, Travnička 1, 72000 Zenica, Bosnia and Herzegovina; 2 Department of Neurosurgery, Cantonal Hospital Zenica, Crkvice 67, 72000 Zenica, Bosnia and Herzegovina; 3 Department of Anatomy, School of Medicine, University of Zenica, Travnička 1, 72000 Zenica, Bosnia and Herzegovina; 4 Department of Genetics and Bioengineering, International Burch University Sarajevo, Francuske revolucije BB, 71000 Sarajevo, Bosnia and Herzegovina; 5 Department of Neurology, Cantonal Hospital Zenica, Crkvice 67, 72000 Zenica, Bosnia and Herzegovina; 6 Department of Histology, School of Medicine, University of Zenica, Travnička 1, 72000 Zenica, Bosnia and Herzegovina; 7 Department of General Medicine, School of Medicine, University of Tuzla, Univerzitetska 1, 75000 Tuzla, Bosnia and Herzegovina; naidasalkovic@gmail.com; 8 Clinic of Neurosurgery, University Clinical Center Sarajevo, Bolnička 25, 71000 Sarajevo, Bosnia and Herzegovina; 9 Department of Neurosurgery, University Hospital Marburg, Baldingerstr., 35033 Marburg, Germany; mirza.po@gmail.com

**Keywords:** glioblastoma, gene therapy, CRISPR, Cas9

## Abstract

This scoping review examines the use of CRISPR/Cas9 gene editing in glioblastoma (GBM), a predominant and aggressive brain tumor. Categorizing gene targets into distinct groups, this review explores their roles in cell cycle regulation, microenvironmental dynamics, interphase processes, and therapy resistance reduction. The complexity of CRISPR-Cas9 applications in GBM research is highlighted, providing unique insights into apoptosis, cell proliferation, and immune responses within the tumor microenvironment. The studies challenge conventional perspectives on specific genes, emphasizing the potential therapeutic implications of manipulating key molecular players in cell cycle dynamics. Exploring CRISPR/Cas9 gene therapy in GBMs yields significant insights into the regulation of cellular processes, spanning cell interphase, renewal, and migration. Researchers, by precisely targeting specific genes, uncover the molecular orchestration governing cell proliferation, growth, and differentiation during critical phases of the cell cycle. The findings underscore the potential of CRISPR/Cas9 technology in unraveling the complex dynamics of the GBM microenvironment, offering promising avenues for targeted therapies to curb GBM growth. This review also outlines studies addressing therapy resistance in GBM, employing CRISPR/Cas9 to target genes associated with chemotherapy resistance, showcasing its transformative potential in effective GBM treatments.

## 1. Introduction

Glioblastoma (GBM) stands out as the predominant and highly aggressive primary brain tumor among adults, constituting approximately 45.2% of all tumors affecting the brain and central nervous system (CNS) [1]. This neoplasm is categorized as a grade IV diffuse astrocytic glioma, distinguished by its elevated cellular density, nuclear abnormalities, microvascular proliferation, necrosis, and invasive characteristics. The molecular features defining GBM encompass mutations in the telomerase reverse transcriptase (TERT) promoter, amplification of the epidermal growth factor receptor (EGFR) gene, and variations in chromosome copy numbers (+7/−10) [2]. Notably, GBM exhibits marked heterogeneity, both in its histological and molecular aspects, contributing significantly to its resistance to therapeutic interventions and dismal prognosis [3].

Primary and secondary GBMs represent discrete disease entities distinguished by disparate genetic pathways, patient demographics, and prognostic outcomes. Primary GBMs arise de novo in older individuals, marked by the upregulation of the epidermal growth factor receptor (EGFR) gene, mutations in the phosphatase and tensin homolog (PTEN), deletions in the cyclin-dependent kinase inhibitor 2A (CDKN2A) gene, and, occasionally, amplification of the mouse double minute 2 homolog (MDM2) gene [1]. On the other hand, secondary GBMs emerge from low-grade or anaplastic astrocytomas, typically occurring in younger patients. They are notably characterized by mutations in the TP53 gene, serving as the earliest detectable genetic alteration. In summary, primary and secondary GBMs are distinct subtypes within the spectrum of GBM, exhibiting contrasting genetic and clinical characteristics [4].

The prevailing standard of care for newly diagnosed GBM involves extensive surgical resection, followed by concurrent chemoradiation employing temozolomide, and subsequent adjuvant temozolomide chemotherapy [5]. Despite the aggressiveness of this therapeutic regimen, its impact on improving survival outcomes is only marginal [1]. Consequently, there exists a compelling demand for novel and efficacious therapeutic approaches for GBM. The past two decades have witnessed a heightened interest in the exploration of targeted agents and immunotherapies for GBM, as reported by Begagić et al. [1]. Regrettably, these endeavors have not yielded a substantive influence on patient survival. GBM remains an incurable affliction, and its management continues to pose one of the most formidable challenges in the realm of neuro-oncology [6]. Furthermore, there is an imperative to formulate therapeutic strategies that are both more potent and less toxic, specifically tailored to address the unique biological characteristics of GBM [7].

As previously mentioned, the presence of genetic mutations, including chromosomal changes such as the loss of chromosomes 10 and 9p, and the gain of chromosomes 7 and 19, suggests the potential utility of gene-oriented therapy as an option in the treatment of GBM [8,9]. One such approach that has garnered significant interest in the last decade across various medical conditions is the Clustered Regularly Interspaced Short Palindromic Repeat CRISPR-associated (Cas) nuclease 9 (CRISPR-Cas9) system [10], which facilitates gene editing technology [11]. CRISPR is recognized as the fastest, cheapest, most versatile, and most reliable gene editing tool available, extensively employed for uncovering genetic alterations, oncogenic targets, and epigenetic regulation. CRISPR-Cas9 stands out as the preferred choice for editing genes or genomes in various cancers, including GBM [12,13,14,15]. Considering the current trend in medical research towards more accessible treatments for diverse pathologies [16], with an aim for broader applicability and treatment options in low- and middle-income countries [17], the potential for gene editing using this method has emerged, even in cases of GBM. There are evident studies employing this method for treating GBM, although the data are dispersed across individual studies. Therefore, the objective of this scoping review is to critically analyze and summarize the diverse advantages and applications of the CRISPR-Cas9 system in the context of precision gene therapy for GBM.

## 2. CRISPR/Cas9 Gene Editing

### 2.1. Brief Historical Overview of CRISPR/Cas9 Technology

CRISPR/Cas9 technology evolved against the backdrop of bacterial immune defense systems, where CRISPR and Cas9 first acted as guardians of innate immunity [18]. The initial pages of CRISPR/Cas9 history highlight the groundbreaking discoveries of scientists, especially Jennifer Doudna and Emmanuelle Charpentier, who clarified the system’s molecular variations. Their revolutionary findings established a path for the scientific community to use CRISPR/Cas9 for precise genome alterations. They won the Nobel Prize in Chemistry in 2020 for their contributions to technology [18]. With the discovery of genome-editing meganucleases in the 1990s, the desire for precision targeting of specific DNA regions became a reality. These molecular scissors, which are found in many organisms, cut particular DNA sequences, allowing for precise replacement, removal, and alteration of DNA with minimal consequences [19].

CRISPR/Cas has gained recognition since its incorporation as a genome editing technology in 2012, outperforming its predecessors in precision editing and attracting interest for possible uses in gene therapy and other applications [20,21,22]. From its modest starting point to its widespread presence in modern laboratories throughout the world, the history of CRISPR/Cas9 is distinguished by the balance of discovery, innovation, and scientific curiosity. The trip of the CRISPR/Cas9 encompasses the understanding of basic biological mechanisms and reactions. Currently, CRISPR/Cas9 is renowned as a flexible and transformational tool for modifying the genome [23].

### 2.2. CRISPR/Cas9 Technology

A thorough examination of the current CRISPR/Cas9 technological environment reveals a dynamic picture of current studies, inventions, and transformational results. CRISPR/Cas9 literature is a dynamic storehouse of information, demonstrating the technology’s ubiquitous effect across many scientific areas. The literature in the realm of medicine attests to CRISPR/Cas9’s rising promise as a therapeutic game changer. Beyond its traditional function in precise genome editing, the technique is now being used to fix infectious mutations, opening up new opportunities for focused treatments [24,25,26,27,28]. The literature reports a hopeful story in which CRISPR/Cas9 emerges as a light for tackling genetic problems that were previously thought unsolvable. 

Xing and Meng [24] presented CRISPR/Cas9 as one of the most powerful tools for the identification of an oncogene, a gene with transformative capabilities that results in a tumor cell. As mentioned in the literature, the technology is known to have potential in different cancer types, from colorectal to acute myeloid leukemia (AML). Jiang et al. [25] proposed that CRISPR/Cas9 technology is the most powerful technology in the world of personalized medicine. Not only is it optimal for removing disease-causing genes, but it is strongly connected with having therapeutic effects by inserting ‘protective’ genes [29]. Wang et al. [26] and Hazafa et al. [27] both mention the case of CRISPR/Cas9-mediated ATG5 knockout that promotes the transformation of ‘bad’ or malignant cells to ‘good’ cells. 

Hsu et al. [28] noted challenges regarding this technology. The problem of pre-existing immunity is still there, along with the potential possibility of removing human pluripotent stem cells. Despite the optimism and promise, the literature emphasizes the ethical concerns and laws and regulations that will accompany the widespread use of CRISPR/Cas9 [30], especially following instances like Chinese researchers manipulating human embryos in 2015 and Dr. He Jiankui’s claim of genome-modified twins in 2018. These events underscore the need for ethical guidelines in CRISPR applications, balancing scientific breakthroughs with societal values and concerns [31,32].

### 2.3. Applications in Gene Therapy

CRISPR/Cas9 emerges as a beacon of hope in the field of personalized medicine, offering tailored interventions based on an individual’s unique genetic profile [27]. CRISPR/Cas9 precision in gene editing ushers in a paradigm shift in therapeutic approaches, with the potential to reduce adverse reactions and enhance the positive results of therapy. The available research, which is rich in case reports and experimental findings, depicts an idea of a future in which CRISPR/Cas9 transforms the therapeutic landscape, providing tailored solutions to previously unresolved genetic challenges.

Within the realm of gene therapy, the revolutionary CRISPR/Cas9 technology has emerged as a powerful tool for precise and targeted modifications to the DNA sequences of cells [33]. This capability enables the correction of genetic defects, opening new avenues for therapeutic interventions. Unlike traditional methods, CRISPR/Cas9 facilitates the deletion, insertion, or modification of specific genes with remarkable accuracy.

Gene therapy holds great promise for treating a variety of genetic disorders, as the CRISPR/Cas9 system allows for the direct correction of harmful mutations within the genome. This approach offers a potential cure for diseases with a known genetic basis, addressing the root cause rather than merely managing symptoms. Initially, viral vector delivery of therapeutic transgenes, mainly for cancer treatment or monogenic diseases, marked the early stages of gene therapy [34]. The CRISPR/Cas system, particularly CRISPR/Cas9, has gained prominence due to its low cost, ease of use, and efficient and precise performance [35]. However, concerns about its delivery using adeno-associated virus (AAV) vectors persist, necessitating exploration of alternative delivery options [36].

Achieving precise genome editing is crucial for the success of CRISPR gene therapy. While homology-directed repair (HDR) pathways have the potential for desired edits, their low efficiency limits their utility for clinical intervention [37]. As Wang et al. [37] noted, strategies to enhance HDR efficiency, such as suppressing the nonhomologous end joining (NHEJ) pathway, have been explored, but challenges remain. Cell cycle stage control and other advancements offer potential avenues to favor templated repair and improve HDR efficiency.

An innovative advancement, the CRISPR base editing system, allows precision gene editing independent of DNA damage response mechanisms [38]. This system, utilizing catalytically inactive dead Cas9 (dCas9) conjugated to deaminase, enables single-nucleotide editing without inducing double-strand breaks. In practical applications, CRISPR/Cas9 has streamlined the generation of transgenic animal models for studying genetic disorders and potential therapeutic interventions [34]. The direct injection of Cas9 protein and transcribed guide RNA into fertilized zygotes allows for heritable gene modifications. This method reduces the time required to generate mutant animal models, making it a cost-effective and efficient approach for in vivo studies [34].

**In summary**, gene therapy, particularly utilizing CRISPR/Cas9 technology, has evolved significantly, offering novel avenues for precision genome editing. Ongoing research and advancements in delivery methods, genome-editing tools, and safety considerations continue to shape the landscape of gene therapy, with the ultimate goal of providing effective and safe therapeutic interventions for a multitude of genetic diseases.

### 2.4. Principles of CRISPR/Cas9 Gene Editing Technology

**Figure 1:**
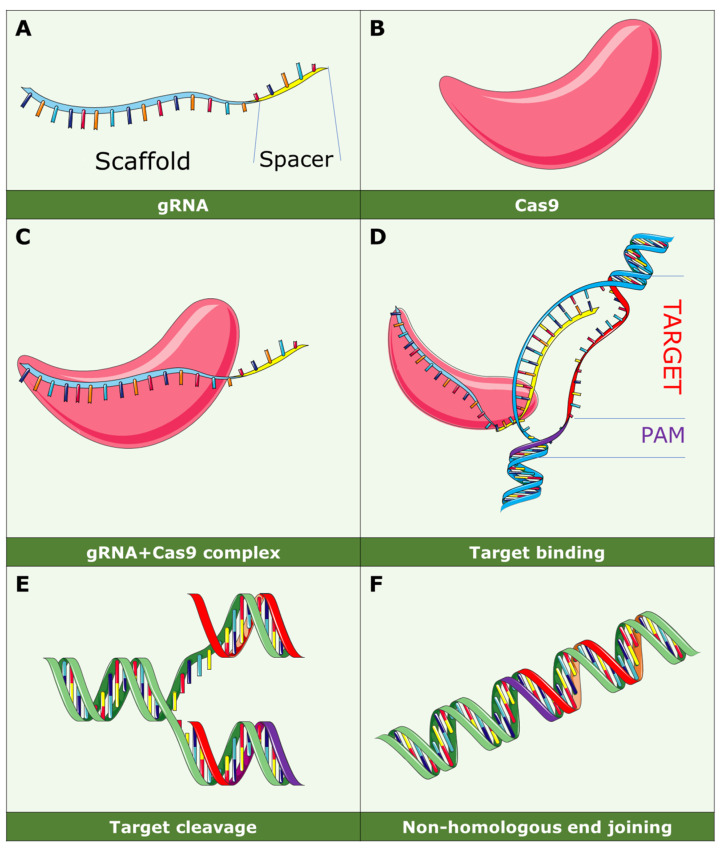
Principles of CRISPR/Cas9 technology. Guided RNA (gRNA) (**A**) and Cas9 (**B**) form a complex (**C**), followed by targeting (**D**) of the specific gene. Subsequently, the gene is edited to remove a specific mutation (**E**), and non-homologous end joining (**F**) takes place.

The CRISPR/Cas9 system is a customizable RNA-guided endonuclease arrangement composed of a Cas enzyme and a guide RNA (gRNA) (Figure 1A) [39]. Essentially, the Cas enzyme (Figure 1B) induces a double-strand break at a specific template location, guided by the sgRNA through Watson–Crick base pairing (Figure 1C). The gRNA comprises two interconnected segments: the CRISPR-RNA (crRNA) and the trans-activating crRNA (tracrRNA) [40]. Designing the crRNA is user friendly, enabling system programmability. The crRNA recognizes the protospacer, corresponding to the target sequence, and the Cas enzyme is triggered by the protospacer adjacent motif (PAM), a short sequence following the protospacer. In the case of Cas9, the double-strand break occurs three bases upstream of the PAM (Figure 1D). Genome editing unfolds during the repair of double-strand breaks initiated by the CRISPR-Cas system, involving two primary repair pathways: homology-directed repair and nonhomologous end joining (NHEJ), with NHEJ being more prevalent in mammalian cells (Figure 1E,F) [41]. The homology-directed repair strategy necessitates a donor template with homology to the contextual sequence, integrating into the double-strand break site for precise genome editing. Despite homology-directed repair predating CRISPR-Cas, the system significantly enhances its efficiency [42]. This method facilitates precise corrections, mutation insertions, or gene insertions, contingent upon co-delivering donor templates. Despite generally low efficiencies and occurrence solely in dividing cells, it amplifies the precision of genome editing. Conversely, NHEJ repairs double-strand breaks in an almost random manner, resulting in minor insertions or deletions at the break site. The outcomes are diverse, often causing frameshift mutations and depleting the target gene function [42,43]. While lacking precision, NHEJ operates without a donor template, functions in both dividing and nondividing cells, and is generally more efficient [44,45].

## 3. CRISPR/Cas9-Mediated GBM Therapy

Distinctive genetic polymorphisms, ionizing radiation exposure, and the impact of chemical carcinogens on brain cells are among the key pathogenic factors driving the development of GBM [46]. Current research is honing in on the promising potential of CRISPR/Cas9 as a cutting-edge gene-editing technology in the realm of immunotherapy for GBM. This innovation is gaining traction in various studies and holds the promise of evolving into a pivotal tool for advancing gene research and engineering strategies in glioma therapy [47,48].

### 3.1. Targeting Specific Genetic Mutations in GBM

Previous research has not provided a clear classification of precise gene therapy for GBM. Based on the study by Begagić et al. [1], it is observed that the main focuses in GBM therapy are the protein kinase pathway, cell-cycle-related mechanisms, and microenvironmental and immunomodulatory targets. In the realm of CRISPR/Cas9 gene editing for GBM, this review categorizes specific gene targets into distinct groups, namely: cell cycle regulation, regulation related to the microenvironment, regulation during cell interphase, and targets related to therapy resistance reduction.

#### 3.1.1. Cell Cycle Regulation

The cell cycle, a meticulously regulated and intricately orchestrated biological process, stands as a fundamental mechanism governing the growth, development, and maintenance of living organisms [1]. Comprising a series of precisely coordinated events leading to cell division, the cell cycle ensures the accurate transmission of genetic information from one generation of cells to the next. In instances where genetic mutations precede this transmission, the altered information is passed on to progeny cells through the process of cell division. This paradigm is particularly relevant to GBM, as disruptions in the cell cycle regulation, stemming from genetic alterations, contribute to the excessive division and proliferation of neoplastic GBM tissue.

**Figure 2:**
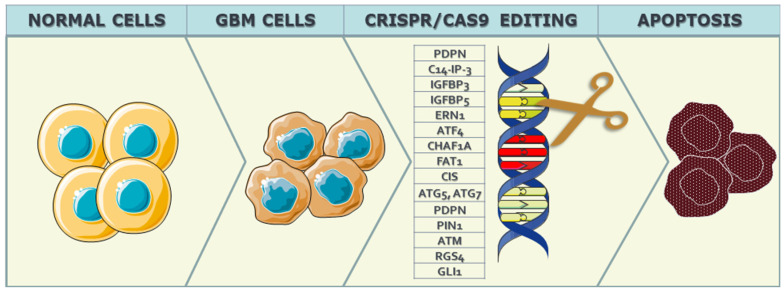
Representation of the CRISPR/Cas9 precise gene editing process. Normal cells refer to cells before neoplastic changes in GBM, followed by gene editing of the target gene to halt the proliferation of malignant cells, slow down the expression of the target gene (knockdown), or completely halt it (knockout).

Given the strict control exerted by genes over the cell cycle, alterations in these genes lead to dysregulation of cell cycle control mechanisms, fostering uncontrolled division and proliferation of neoplastic GBM cells. Several mutations associated with GBM malignancy have been identified, including those affecting genes such as Epidermal Growth Factor Receptor (EGFR), Erb-B2 Receptor Tyrosine Kinase 2 (ERBB2), Isocitrate Dehydrogenase 1 (IDH1), Neurofibromin 1 (NF1), Phosphatidylinositol-4,5-Bisphosphate 3-Kinase Catalytic Subunit Alpha (PIK3CA), Phosphoinositide-3-Kinase Regulatory Subunit 1 (PIK3R1), and Phosphatase and Tensin Homolog (PTEN), among others. The application of CRISPR/Cas9 technology seeks to intervene in the cell cycle of neoplastic cells, aiming to induce apoptosis or autophagy in these aberrant cells, as can be seen in Figure 2.

The promotion of apoptosis and autophagy through CRISPR-Cas9 technology has been substantiated through research conducted on GBM cellular lines and in vitro models. Various target genes have been investigated in this context, including Podoplanin (PDPN), C14orf166 (C14-IP-3), Insulin-Like Growth Factor Binding Protein 3 and 5 (IGFBP3 and IGFBP5), Endoplasmic Reticulum To Nucleus Signaling 1 (ERN1), Activating Transcription Factor 4 (ATF4), Autophagy Related 5 (ATG5), Chromatin Assembly Factor 1 Subunit A (CHAF1A), FAT Atypical Cadherin 1 (FAT1), Cytokine-Inducible SH2-Containing Protein (CIS), Autophagy Related 7 (ATG7), and PIN1 (Peptidylprolyl Isomerase NIMA-Interacting 1) (Table 1).

In the context of CRISPR-Cas9 technology, it is essential to distinguish between knockout, knockdown, and knock-in strategies. While knockout involves the complete elimination of a targeted gene (Figure 1), exemplified by the knockout of the FAT1 gene in GBM cells to enhance susceptibility to apoptosis, knockdown selectively reduces the expression of a gene, as demonstrated in the PDPN gene knockdown study, aiming to modulate apoptosis and cell proliferation.

Wang et al. [49] employed a knockdown strategy targeting the PDPN gene to intricately modulate apoptosis and cell proliferation. This approach specifically focused on manipulating PDPN surface-membrane cell molecules. The presence of mutations in PDPN aligns with the malignancy, aggressiveness, and invasiveness of GBM. The identified association between PDPN overexpression and the facilitation of macrophage M2 polarization and neutrophil degranulation underscores the immunomodulatory impact of PDPN within the tumor microenvironment. The observed shift towards M2 polarization of macrophages and the induction of neutrophil degranulation collectively indicate a coordinated effort by PDPN to establish an environment conducive to immune evasion and tumor progression [49]. This nuanced insight challenges conventional perspectives on PDPN, positioning it as a pivotal orchestrator in shaping an immunosuppressive milieu specifically within IDH wildtype gliomas. These findings highlight the complex and multifaceted role of PDPN in influencing not only the cellular aspects of apoptosis and proliferation but also the intricate immunological dynamics within the tumor microenvironment. Employing a strategy that initiated apoptosis through the Unfolded Protein Response (UPR) mechanism, IGFBP3 and IGFBP5 were targeted for knockout, shedding light on their influence on cell cycle dynamics [51]. Further insights were contributed by investigating ATG5 knockout, revealing its dual impact on apoptosis and autophagy and providing understanding within the framework of cell cycle processes associated with GBM [52]. Also, CHAF1A was subjected to CRISPR-Cas9 knockout, dissecting its consequences on the AKT/FOXO3a/Bim pathway and influencing proliferation and DNA repair mechanisms integral to cell cycle regulation [53]. Targeting the FAT1 gene for knockout revealed its active involvement in apoptosis through the Death-Inducing Signaling Complex (DISC), contributing valuable insights into the understanding of key molecular players that impact the cell cycle during GBM progression [54]. Shifting the focus to the CIS gene, investigations aimed to unravel its role in NK-cell activation and apoptosis, establishing a link between immune responses and the intricate molecular mechanisms that influence the cell cycle [55]. Furthermore, examinations delved into the consequences of knockout for ATG5 and ATG7 on the autophagosome membrane [56]. This shed light on the potential significance of autophagy in regulating the cell cycle in the context of GBM. PIN1, targeted for knockout, offered a comprehensive exploration of its multifaceted role in influencing the cell cycle within GBM development, encompassing aspects of apoptosis, migration, and cell cycle progression. The knockout study involving ATM, PTEN, p85α, and XIAP genes uncovered their roles as tumor suppressors, advancing our comprehension of the intricate regulatory mechanisms that govern the cell cycle in GBM [58]. Further investigations focused on RGS4 knockout explored its impact on apoptosis through G protein signaling, expanding the repertoire of molecular targets with potential therapeutic implications in the context of cell cycle regulation. Lastly, the exploration of GLI1’s involvement in apoptosis through the PI3K/Akt pathway contributed to the elucidation of critical signaling pathways that influence the cell cycle in the pathophysiology of GBM [60].

In summary, the intricate landscape of CRISPR-Cas9 applications in GBM research unveils a spectrum of gene manipulation strategies, each offering unique insights into the regulation of apoptosis, cell proliferation, and immune responses within the tumor microenvironment. From knockout endeavors targeting genes such as FAT1, ATM, PTEN, and GLI1 to deciphering the roles of PDPN, IGFBP3, and CHAF1A through knockdown and knock-in approaches, the studies presented here collectively broaden our understanding of the complex molecular orchestration shaping GBM progression. These findings not only challenge conventional perspectives on specific genes but also underscore the potential therapeutic implications of manipulating key molecular players in the intricate web of cell cycle dynamics. As the field continues to unravel the complexities of CRISPR-Cas9 technology in the context of GBM, these insights hold promise for advancing targeted therapeutic interventions and refining our approach to combating this formidable malignancy.

#### 3.1.2. Cell-Interphase-Related Targets

Exploring the realm of CRISPR/Cas9 gene therapy in the context of GBMs, researchers have delved into a diverse array of cell-interphase-related targets to unravel the intricacies of gene regulation during critical phases of the cell cycle. The focus on cell interphase, the period between cell divisions encompassing G1, S, and G2 phases, is crucial in understanding the dynamics of GBM progression and identifying potential therapeutic avenues [61].

In recent studies, various genes have been targeted using CRISPR-Cas9 gene editing technology to elucidate their roles in cell proliferation and related functions. Fierro et al. [62] focused on PD-L1, employing a knockout strategy to investigate its impact on proliferation, invasion, and macrophage polarization. Lumibao et al. [63] targeted CHCHD2, aiming for knockout to understand its influence on mitochondrial respiration, glutathione status, and cell growth inhibition, particularly in the context of EGFRvIII. Toledano et al. [64] explored Plexin-A2 through knockout, shedding light on its involvement in cytoskeletal organization, cell flattening, and cell cycle arrest, with a focus on β-galactosidase, MAPK, and FARP2. Gallo et al. [13] delved into the knockout of 14-3-3β, unraveling its effects on proliferation, spheroid formation, and interactions with Bad, FBI1, Raf-1, and Cdc25b. Additionally, Meng et al. [65] investigated CDK7, employing a knockout strategy to understand its role in cellular growth. Guda et al. [59] targeted RGS4 for knockout, exploring its influence on MMP2 and proliferation. Zhang et al. [66] utilized a knockdown strategy for Nanos3, examining its effects on CD133, Oct4, and its implications for proliferation, migration, and chemoresistance. Godoy et al. [67] employed knockdown of NRF2, investigating its role in self-renewal and cell proliferation, particularly in relation to SOD. Zhang et al. [68] focused on Dazl, utilizing knockout to study its involvement in the CD133/Oct4/Nanog/Sox2 regulatory axis and its impact on proliferation. Lastly, Liu et al. [69] explored ERβ through knockout, elucidating its effects on proliferation and apoptosis by targeting ERβ1, ERβ2, ERβ3, ERβ4, ERβ5 (exon 8), mTOR, and STAT-3 (Table 2).

Cell renewal studies employing CRISPR/Cas9 technology have investigated specific genes and their roles in regulating crucial aspects of this process. In the work by Bulstrode et al. [70], the focus was on Foxo3, utilizing a knockdown approach to assess its impact on differentiation. Specifically, the study targeted FOXG1, SOX2, EGFR, and EGFRvIII in order to delineate their involvement in cell renewal pathways. Saenz-Antonanzas et al. [71] explored the role of SRR2 through deletion, aiming to understand its influence on self-renewal capacity with a particular emphasis on SOX2. Additionally, Song et al. (2019) investigated SRSF3 using knockout techniques, unraveling its significance in glioma-associated alternative splicing processes involving SR proteins.

Studies targeting cell migration through CRISPR/Cas9 technology have provided valuable insights into the molecular underpinnings of this crucial cellular process. Ogawa et al. [73] focused on TP53, employing recombination techniques to explore its influence on migration. Smolkin et al. [74] investigated NRP2, Plexin-A4, Plexin-D1, and Semaphorin-3C through knockout strategies, shedding light on their roles in regulating migration processes. Prolo et al. [75] delved into MAP4K4 using knockout, elucidating its impact on both migration and invasion. Wang et al. [76] explored the knockout of BRG1, revealing its involvement in migration, proliferation, and resistance to TMZ. Shao et al. [77] targeted PIK3CD along with PAK3 and PLEK2 for knockout, unraveling their roles in migration and invasion. Chen et al. [78] investigated THBS1 and TNF through knockout, shedding light on their contributions to proliferation and migration. Ozyerli-Gokna et al. [79] focused on ASH2L and SET1/MLL, employing knockout to understand their roles in both proliferation and migration. Nieland et al. [80] targeted miR21 and SOX2 through knockout, providing insights into their contributions to migration, invasion, and proliferation. Lastly, Uceda-Castro et al. [81] investigated GFAP, GFAPα, and GFAPδ through knockout, revealing their involvement in invasion processes.

**In summary**, the comprehensive exploration of CRISPR/Cas9 gene therapy in the intricate landscape of GBMs has yielded significant insights into the regulation of crucial cellular processes, spanning cell interphase, renewal, and migration. Through precise targeting of specific genes, researchers have unraveled the complex molecular orchestration governing cell proliferation, growth, and differentiation during critical phases of the cell cycle. The investigations into cell renewal shed light on the roles of Foxo3, SRR2, and SRSF3 in influencing self-renewal capacity and alternative splicing processes. Furthermore, studies elucidating the molecular underpinnings of cell migration, targeting genes such as TP53, NRP2, MAP4K4, BRG1, PIK3CD, THBS1, PD-L1, ASH2L, SET1/MLL, miR21, SOX2, and GFAP, have provided valuable insights into the regulation of migration, invasion, and proliferation in the context of GBMs.

#### 3.1.3. Microenvironmental CRISPR/Cas9 Targets in GBM Cells

**Figure 3:**
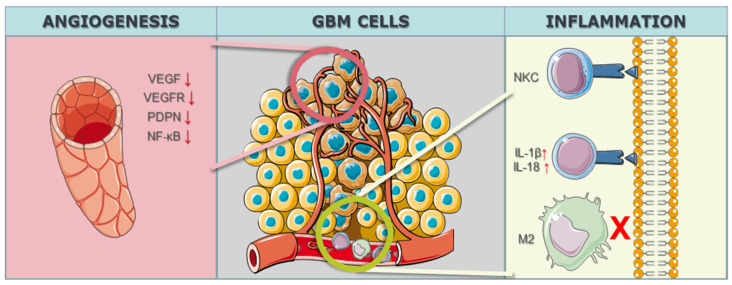
CRISPR/Cas9-related targets within the microenvironment. Angiogenesis is attenuated or entirely halted through the reduction (marked as ↓) in VEGF (Vascular Endothelial Growth Factor) and its receptor (VEGFR), as well as PDPN and NF-kB. CRISPR/Cas9 technology has effectively directed an inflammatory response against GBM cells in in vitro models, activating Natural Killer Cells (NKC) and increasing IL-1 and IL-18 (marked as ↑), inducing pyroptosis in the cells. Inhibiting (marked as X) the actions of M2 macrophages has proven effective in the immune regulation of GBM.

In the realm of GBM research, the intricate modulation of the tumor microenvironment, particularly in the context of angiogenesis, has become a focal point for therapeutic interventions. The study by Han et al. [82] targeted the Notch1 gene, employing a knockdown strategy to address hypoxia, angiogenesis, and tumor growth. Notch1 is known for its involvement in diverse cellular processes, and its modulation in the study aimed at disrupting key pathways associated with angiogenesis, a hallmark feature of GBM progression. By utilizing CRISPR/Cas9 technology to downregulate Notch1 expression, the study sought to unravel the intricate interplay between hypoxia, angiogenesis, and the overall growth dynamics of GBM malignant cells. Eisemann et al. [83] delved into the role of PDPN, employing a knockout strategy to investigate its influence on the maturation and integrity of the developing vasculature in the murine brain. PDPN, when interacting with C-type lectin-like receptor 2 on platelets, has been implicated in mediating vascular development. By utilizing CRISPR/Cas9 to knockout PDPN, the study aimed to disrupt the finely tuned mechanisms governing vasculature maturation, potentially impeding the vascular support crucial for GBM growth and progression. The targeted gene PDPN serves as a molecular focal point, shedding light on its intricate involvement in orchestrating the vascular microenvironment within the context of GBM. Szymura et al. [84] explored the role of DDX39B in regulating the extracellular extracellular matrix (ECM) and promoting angiogenesis through the NF-κB pathway. By employing a knockdown strategy, the study aimed to decipher the contributions of DDX39B in modulating the complex network of signals involved in angiogenesis and ECM regulation. The NF-κB pathway, known for its involvement in various cellular processes, including inflammation and angiogenesis, was specifically targeted to understand its role in the GBM microenvironment. The study adds depth to our understanding of how specific genes can be manipulated to influence the intricate balance of proangiogenic factors in the context of GBM. Continuing the exploration of angiogenesis-related genes, Lu et al. [85] investigated the genes BIG1 and BIG2, targeting VEGF through a knockdown approach in 2019. VEGF is a key player in angiogenesis, promoting the formation of new blood vessels to sustain tumor growth. By employing CRISPR/Cas9 to knock down BIG1 and BIG2 and subsequently reduce VEGF levels, the study aimed to disrupt the angiogenic signals that contribute to the vascularization of GBM tumors. The modulation of these specific genes provides insights into the intricate regulatory mechanisms underlying angiogenesis and presents potential avenues for therapeutic interventions aimed at curbing the growth and progression of GBM through microenvironmental control (Figure 3) (Table 3).

Table 3, alongside angiogenesis-related targets, delineates additional microenvironment-related targets with a focus on inflammation within the context of GBM. Nakazawa et al. [55] investigated the CIS gene, employing CRISPR/Cas9 knockout to enhance the effects of Natural Killer Cells (NKCs) and bolster the inflammatory response against GBM. Similarly, Wei et al. [87] targeted the OPN gene for knockout, resulting in the reduction in M2 macrophages and a concurrent elevation in T-lymphocyte effector activity, thereby influencing the inflammatory landscape within the tumor microenvironment. Additionally, Chen et al. [88] utilized CRISPR/Cas9 knockdown to modulate the AIM2 gene, leading to a downregulation of interleukins IL-1β and IL-18 and inducing pyroptosis, an inflammatory programmed cell death.

**In summary**, the collective findings from these studies underscore the potential role of CRISPR/Cas9 technology in unraveling the complex dynamics of the GBM microenvironment. Through precise manipulation of key genes involved in angiogenesis, such as Notch1, PDPN, DDX39B, and VEGF-related genes (BIG1 and BIG2), researchers have gained insights into the molecular intricacies governing vascularization, providing potential targets for therapeutic intervention. The multifaceted impact of CRISPR/Cas9 is further demonstrated in Table 3, where the focus shifts to inflammation within the GBM microenvironment. By targeting genes like CIS, OPN, and AIM2, these studies leverage CRISPR/Cas9 to enhance Natural Killer Cell effects, modulate M2 macrophages, and induce pyroptosis, collectively contributing to the immune regulation of GBM. The versatility of CRISPR/Cas9 gene editing in manipulating these microenvironment-related targets presents promising avenues for developing targeted therapies to curb the growth and progression of GBM, showcasing its potential as a transformative tool in the quest for effective GBM treatments.

### 3.2. Contribution of CRISPR/Cas9 Technology in Alleviating Therapy Resistance of GBM

The provided Table 4 outlines several studies investigating the application of CRISPR/Cas9 technology to address therapy resistance in GBM. In the study by Wu et al. [89], the ALDH1A3 gene was targeted for knockdown, focusing on ALDHs to mitigate resistance to temozolomide (TMZ), a common chemotherapy agent. Similarly, Han et al. [90] in 2023 employed knockdown targeting the MGMT gene, specifically addressing TMZ resistance. Tong et al. [91], also in 2023, utilized knockdown techniques targeting the MUC1 gene, associated with EGFRvIII, to overcome TMZ resistance. Liu et al. [92] focused on the GSS gene, employing knockout to address radiotherapy resistance, particularly in the context of Angiopep-2. Rocha et al. [93], in 2020, targeted multiple genes, including MSH2, PTCH2, CLCA2, FZD6, CTNNB1, and NRF2, focusing on transmembrane proteins to counter TMZ resistance through CRISPR/Cas9 knockout. Lastly, Yin et al. [94] targeted the HPRT1 gene for knockout, with a focus on AMPK, aiming to alleviate TMZ resistance.

CRISPR/Cas9 gene editing has proven successful in addressing treatment resistance in GBM. Wu et al. [89] targeted ALDH1A3, achieving a significant impact on TMZ resistance, particularly at dosages ≤300 μM. Han et al. [90] successfully used knockdown of MGMT to sensitize GBM cells to TMZ treatment. Additionally, Tong et al. [91] demonstrated success by targeting MUC1 with knockdown, revealing its role in DNA damage repair during chemotherapy and radiation.

However, not all interventions yielded successful outcomes. Rocha et al. [93] targeted MSH2, PTCH2, CLCA2, FZD6, CTNNB1, and NRF2 for TMZ resistance through knockout, but silencing the top three genes did not sensitize GBM cells to TMZ.

## 4. Efficacy and Safety Considerations

CRISPR/Cas9 is a powerful tool for genome editing with great potential for therapeutic development, enhancing cancer immunotherapy by regulating CAR T cell cytotoxicity and shedding light on genetic modifications of tumor cells. However, its efficacy and long-term safety remain primary concerns in clinical application. Off-target editing events, large DNA deletions, and rearrangements are major safety concerns that need to be addressed in preclinical studies. The high frequency of off-target effects and the potential for unintended consequences of on-target activity are significant limitations for safe and efficient clinical use. In addition, CRISPR/Cas9 raises concerns for immunogenic toxicity, and the delivery of the machinery to the right cell target in the body is still a limitation. Understanding and mitigating these safety issues are crucial for the successful clinical application of CRISPR/Cas9 technology. Cas9 protein and sgRNA inhibit PD-1 expression in EvCAR T cells show an inhibitory effect on EGFRvIII GBM cells without influencing other checkpoint receptors. In vivo results are yet to be determined. Gene editing with CRISPR-Cas9 has the power to enact multiple checkpoint signal disruptions, enhancing antitumor utility. One of the risks of CRISPR therapy is the possibility of off-target mutagenesis. More clinical studies are needed to properly evaluate the efficacy and safety of CRISPR/Cas9.

p53, recognized as the guardian of the genome, plays a pivotal role in detecting DNA damage and orchestrating cellular responses such as halting cell division and inducing programmed cell death [95]. This innate defense mechanism serves as a barrier against cancer and complications arising from DNA damage. In the context of CRISPR gene editing, a process involving the cleavage of both DNA strands, an inherent challenge emerges [95]. The manipulation may trigger a p53 response, leading edited cells to be identified as damaged and subsequently eliminated. This response, while a protective measure, can diminish the efficiency of the gene editing process. Complicating matters, the interaction between p53 and gene editing becomes intricate as cells that evade CRISPR-induced alterations may do so due to impaired p53 functionality [95,96]. Consequently, these cells may exhibit reduced capacity to detect DNA damage or label cells for programmed death. This scenario introduces a potential complication wherein the gene editing procedure could inadvertently favor the survival of cell populations with compromised p53, rendering them less capable of maintaining genomic stability. This compromised stability increases the likelihood of accumulating additional mutations, thereby elevating the risk of developing malignancies [97,98].

In the pursuit of modifying GBM biology through CRISPR/Cas9 gene editing, successful outcomes have been achieved in various studies. Fierro et al. [62] successfully employed a knockout strategy targeting PD-L1, resulting in a notable 64% reduction in PD-L1 protein levels in U87 cells. Lumibao et al. [63] utilized CRISPR Cas9 knockout of CHCHD2 in EGFRvIII-expressing U87 cells, leading to altered mitochondrial respiration, glutathione status, and decreased cell growth and invasion. Toledano et al. [64] achieved positive results through Plexin-A2 knockout, revealing that Plexin-A2’s proproliferative effects are mediated by FARP2, FYN, and the GTPase activating (GAP) domain in its intracellular domain. Furthermore, Guda et al. [59] targeted RGS4 with a knockout approach, resulting in reduced proliferation. Zhang et al. [66] employed a knockdown strategy on Nanos3, leading to reduced proliferation, migration, and invasion of GBM cells in vitro, increased sensitivity to DOX and TMZ, and inhibited tumor growth in vivo.

Conversely, some therapeutic interventions did not yield the anticipated success, leading to unexpected outcomes. For instance, Ogawa et al. [73] targeted TP53 through recombination, but the effects on migration were not specified. Smolkin et al. [74] utilized CRISPR/Cas9 knockout of NRP2, Plexin-A4, Plexin-D1, and Semaphorin-3C, revealing that Sema3D and Sema3G could not transduce signals without neuropilins. Prolo et al. [75] targeted MAP4K4, resulting in a 41% reduction in invasion compared to controls. Wang et al. [76] achieved positive outcomes through BRG1 knockout, inhibiting GBM cell migration and invasion and sensitizing cells to TMZ. However, in some cases, knockout or knockdown did not lead to the expected results, emphasizing the complexity of GBM biology and the need for further exploration in developing effective therapeutic strategies.

In the realm of CRISPR/Cas9 gene editing for GBM, several studies have reported successful outcomes in modulating angiogenesis. Han et al. [82] employed a knockdown approach to target Notch1, revealing that xenografts with Notch1 downregulation exhibited a significant increase in volume, reaching six times the starting volume in 18.3 days, compared to control xenografts, which took 13.4 days. Eisemann et al. [83] targeted PDPN using a knockout strategy, observing similar rates of proliferation, apoptosis, angiogenesis, and invasion in control and podoplanin-deleted tumors. Szymura et al. [84] focused on DDX39B with a knockdown approach, demonstrating that CRISPR-mediated DDX39B depletion increased p65 phosphorylation and rendered U87 cells highly resistant to TMZ. Additionally, Lu et al. [85] utilized knockdown of BIG1 and BIG2 to significantly decrease VEGF mRNA and protein levels in GBM U251 cells and human umbilical vein endothelial cells (HUVECs). Lee et al. [86] targeted ANGPT2 with a knockout approach, and the treatment with the agonistic anti-Tie2 antibody, 4E2, resulted in vascular normalization throughout GBM tissues.

Conversely, in addressing inflammation, some therapeutic interventions did not yield the expected success. Nakazawa et al. [55] targeted CIS with a knockout approach to enhance natural killer cells (NKCs) effects, but the NK dCIS group exhibited prolonged overall survival compared to the NK mock group, leading to unexpected outcomes. Additionally, Wei et al. [87] employed a knockout strategy for osteopontin (OPN), intending to reduce M2 macrophages and elevate T-lymphocyte effector activity. While OPN deficiency in innate immune or glioma cells reduced M2 macrophages, the observed elevation in T cell effector activity infiltrating the glioma was an unanticipated outcome. Furthermore, Chen et al. [88] used a knockdown approach to target AIM2 for pyroptosis, but AIM2 immunoreactivity concentrated in the tumor core, showing unexpected results in the absence of PCNA immunodetection. These instances emphasize the complexity of the biological responses in GBM to CRISPR/Cas9 interventions and the necessity for further investigations to refine therapeutic strategies.

## 5. Future Directions and Considerations

### 5.1. Ethical and Regulatory Considerations

Several considerations remain paramount in the application of the CRISPR/Cas9 system in routine clinical practice for GBM treatment. A critical concern is the potential elevation in the incidence of de novo mutations in target cells, particularly when employing viral Cas9 nucleases [99,100]. The imprecision of the CRISPR/Cas9 system may result in unintended DNA sequences, giving rise to novel mutations capable of inducing cell death or cellular transformation into neoplastic entities [101,102,103]. Additionally, the efficient and secure delivery of CRISPR-Cas9 to targeted cells presents a significant challenge, given the large size of Cas9 and its difficulty in encapsulation within delivery systems. Consequently, the engineering of precision-based transportation systems capable of traversing the formidable blood–brain barrier, such as nanoparticles or exosomes, becomes imperative [104,105]. Moreover, the regulatory landscape surrounding the patenting of these delivery systems poses an additional obstacle. The ethical quandary arises from the potential prioritization of economic gains for biotechnological companies and the expedited development of novel immunotherapeutic techniques over the benefits accrued by patients.

### 5.2. Possibilities for Personalized Gene Editing in GBM

The advancement of personalized cancer treatment, tailoring drugs and protocols based on individual genomic or epigenomic mutations specific to tumor cells, holds promise for diverse cancer types. Notably, studies on GBM patients reveal a spectrum of mutations in tumor cells, including TP53, INK4A/ARF, PTEN, or NF-1 [12,106]. These mutations contribute to GBM development and its proliferation within brain tissue [107]. Surgical samples from GBM patients exhibit significant histological diversity, indicative of reduced efficacy in conventional therapeutic approaches, leading to the disease’s poor prognosis and aggressive nature [108]. Personalized medicine, designed around drugs engineered for a patient’s molecular alterations and identified tumor cell mutations, offers a promising avenue for individuals with GBM. Novel GBM therapies should address inter-tumoral heterogeneity and the specific immunological characteristics of these cells. However, uncertainties persist regarding clonal heterogeneity in GBM samples, posing a significant challenge in developing targeted drugs. A multi-agent approach may present a potential method to prevent GBM recurrence by selectively eliminating specific tumor-initiating clones, but further research is required for confirmation [109].

### 5.3. Limitations and Challenges of the CRISPR/Ca9 Therapy

Since 2015, numerous clinical studies have explored the CRISPR/Cas9 system for gene editing, showcasing promising progress in its application for GBM patients [46]. However, persistent challenges remain, with the primary impediment being the substantial size of the Cas9 nuclease, hindering efficient delivery. Novel studies aim to engineer an effective delivery system for CRISPR-Cas9, utilizing lipid nanoparticles to inhibit further tumor development following single or double applications and simultaneously addressing the low permeability of the blood–brain barrier (BBB) for conventional cancer drugs [110,111].

Despite the potential of CRISPR/Cas9 as a novel GBM treatment strategy, a major limitation lies in its off-target effects on bystander cells due to the lack of absolute specificity in the utilized DNA and RNA for the targeted gene. A proposed solution involves targeting different sequences in specific genes using several different short interfering RNA (siRNA), as well as combining CRISPR-Cas9 with siRNA to mitigate these off-target effects [102]. Furthermore, the precision of CRISPR/Cas9 gene editing in GBM currently lacks accuracy [104]. The imprecise cutting of DNA sequences by the Cas9 nuclease can introduce new mutations in normal cells, promoting GBM growth [105]. Additionally, the Cas9 nuclease, typically derived from bacteria, may be recognized as a foreign body by the host’s immune system, potentially triggering a hazardous immune reaction [106]. Consequently, repeated dosing of Cas9 could induce immune-related side effects, leading to the termination of treatment for the patient. This underscores the need for engineered delivery systems that require minimal administration and can efficiently treat GBM after a single exposure to the drug.

### 5.4. Toxicology of CRISPR/Cas9 in GBM Therapy

In addition to the limitations and challenges previously discussed, it is crucial to consider the toxicological implications of CRISPR/Cas9 technology, particularly in the context of GBM therapies. As of now, the understanding of CRISPR/Cas9 technology’s potential negative effects on humans, particularly concerning the central nervous system, remains limited and largely theoretical [23,112]. The most significant concerns center around off-target effects, where unintended genetic mutations could occur due to CRISPR/Cas9 editing genes other than the intended targets. Such off-target mutations, if they affect the central nervous system, could potentially disrupt normal neuronal function or lead to neurological disorders.

Additionally, the immune response to the bacterial-origin Cas9 protein is a concern, as it could induce inflammatory or immune-related complications within the central nervous system [113]. These risks underline the complexity and potential unpredictability of applying CRISPR/Cas9 technology in human neurological contexts [114].

Beyond neurotoxicity, CRISPR/Cas9 might exhibit systemic toxic effects [115]. The immunogenicity of the Cas9 protein, typically derived from bacteria, poses a risk of eliciting an adverse immune response, potentially leading to systemic inflammation or autoimmune reactions [95,116,117]. The repeated administration of CRISPR components could exacerbate these effects, necessitating the development of delivery systems that minimize immune recognition [118,119].

Addressing these toxicological challenges is essential for the safe application of CRISPR/Cas9 in GBM treatment. Recent advancements in targeted delivery systems aim to reduce off-target effects and immunogenic responses. For instance, lipid-nanoparticle delivery systems are being explored to enhance the precision and reduce the systemic exposure of CRISPR components [120,121].

**In summary**, while the potential of CRISPR/Cas9 in GBM therapy is significant, a comprehensive understanding of its toxicological profile is imperative. Future research and clinical applications must carefully weigh the therapeutic benefits against potential neurotoxic and systemic effects to ensure the safe and effective management of GBM using gene-editing technologies. Furthermore, the long-term implications of CRISPR/Cas9 gene editing in humans, especially in the nervous system, are not fully understood, primarily due to the novelty of the technology and the ethical and regulatory constraints on human experimentation. The lack of extensive human data limits the ability to conclusively determine the safety and efficacy of CRISPR/Cas9 for neurological applications. Ethical considerations are particularly pertinent in gene editing research, which has led to stringent regulations and careful scrutiny of CRISPR/Cas9 applications in humans. Therefore, while CRISPR/Cas9 holds great promise for treating various diseases, including neurological conditions, comprehensive research and clinical trials are essential to fully ascertain its safety and potential adverse effects in human applications.

## 6. Conclusions

In conclusion, this scoping review provides a comprehensive overview of the promising applications of CRISPR/Cas9 gene editing in addressing the intricate challenges associated with GBM. The systematic categorization of gene targets into distinct groups reveals the multifaceted nature of CRISPR-Cas9 interventions, shedding light on critical cellular processes, including apoptosis, cell proliferation, and immune responses within the GBM microenvironment. The studies presented challenge traditional understandings of specific genes and underscore the therapeutic potential of manipulating key molecular players in cell cycle dynamics. Notably, the exploration of CRISPR/Cas9 gene therapy in GBMs unveils significant insights into the regulation of cellular processes, providing a nuanced understanding of cell interphase, renewal, and migration dynamics. By precisely targeting specific genes, researchers unravel the molecular orchestration governing cell proliferation, growth, and differentiation during crucial phases of the cell cycle.

Furthermore, the collective findings emphasize the transformative potential of CRISPR/Cas9 technology in unraveling the complexities of the GBM microenvironment, presenting promising avenues for developing targeted therapies to mitigate GBM growth and progression. The studies addressing therapy resistance through CRISPR/Cas9 interventions offer a strategic approach to overcome challenges associated with chemotherapy resistance, showcasing the adaptability and effectiveness of CRISPR/Cas9 as a transformative tool in the pursuit of viable GBM treatments. Despite the remaining obstacles, the evolving landscape of CRISPR/Cas9 applications in GBM research holds great promise for advancing precision gene therapy and refining therapeutic interventions for this formidable malignancy.

## Figures and Tables

**Table 1:** Overview of studies investigating CRISPR/Cas9 gene editing for the purpose of regulating the cell cycle in GBM malignant cells.

Reference Year	Targeted Gene	Targeted Molecules or Focus	Targeted Function	CRISPR-Cas9 Gene Editing	Therapy Efficiency or Outcome
Wang et al. [49] 2023	PDPN	PDPN	Apoptosis; Cell proliferation	Knockdown	PDPN may contribute to GBM immune microenvironment, with AUCs for 1-, 3-, and 5-year OS at 0.887, 0.916, and 0.870.
Nayak et al. [50] 2018	C14-IP-3	EGFR, AKT, TP53, RAF1	Regulation of proliferation and invasion	CRISPRa	miR-134 targets EGFR and RAF1, confirmed with luciferase assay.
Rodvold et al. [51] 2019	IGFBP3, IGFBP5, ERN1, ATF4.	IGFBP3, IGFBP5, IRE1α, ATF4.	Apoptosis via UPR	Knockout	Nonresponder phenotype is linked to UPR gene expression, particularly ERN1 and ATF4. CRISPR-deletion of ERN1, IGFBP3, and IGFBP5 in U251 cells enhances responsiveness to 12ADT.
Thi Vu et al. [52] 2018	ATG5	ATG5	Apoptosis, autophagy	Knockout	Ca2+ mobilization compounds combined with autophagy inhibition may be a novel therapy for GBM.
Peng et al. [53] 2018	CHAF1A	AKT, FOXO3a, Bim	Proliferation and DNA repair	Knockout	CRISPR/Cas9 knockout of CHAF1A inhibits FOXO3a transactivity, upregulating Bim and caspase cleavage.
Kranz et al. [54] 2014	FAT1	Caspase-8	Apoptosis via Death-Inducing Signaling Complex (DISC)	Knockout	FAT1 knockout with CRISPR/Cas9 increases susceptibility to death receptor-mediated apoptosis.
Nakazawa et al. [55] 2023	CIS (deleted NKCs)	IFNɤ TNF	NK cells activation; apoptosis	Knockout	CIS deletion enhances NKC-mediated anti-tumor effects in allogeneic GBM.
Zielke et al. [56] 2018	ATG5 ATG7	ATG5 ATG7	Autophagosome membrane	Knockout	Loperamide, pimozide, and STF-62247 induce ATG5- and ATG7-dependent cell death in GBM, preceded by autophagy induction.
Wang et al. [49] 2023	PDPN	PDPN	Apoptosis; Cell proliferation	Knockdown	
Maggio et al. [57] 2023	PIN1	PIN1 enzyme	Apoptosis, migration, cell cycle progression	Knockout	PIN1 deletion in GBM diminishes active NF-κB, reducing il-8 and htert gene transcription.
Reem et al. [58] 2019	ATM, PTEN, p85α, XIAP	PI3K, PIKK, p110α	Tumor suppressors	Knockout	ATM’s novel role in autophagy regulation via XIAP interaction is speculated.
Guda et al. [59]	RGS4	MMP2	Apoptosis (G protein signaling)	Knockout	Silencing RGS4 in GSC20 and GSC28 cells demonstrates anticancer effects, establishing RGS4 as a promoter of invasive behavior in GSCs.
Ranjan et al. [60] 2017	GLI1	PI3K/Akt	Apoptosis	Knockout	Penfluridol treatment suppresses Akt phosphorylation, reduces GLI1, OCT4, Nanog, Sox2 expression, inhibiting tumor growth.

C14-IP-3—Chromosome 14 Internal Promoter 3; EGFR—Epidermal Growth Factor Receptor; AKT—Protein Kinase B; TP53—Tumor Protein P53; RAF1—Raf-1 Proto-Oncogene, Serine/Threonine Kinase; IGFBP3—Insulin-Like Growth Factor Binding Protein 3; IGFBP5—Insulin-Like Growth Factor Binding Protein 5; ERN1—Inositol-Requiring Enzyme 1 Alpha; ATF4—Activating Transcription Factor 4; ATG5—Autophagy-Related 5; CHAF1A—Chromatin Assembly Factor 1 Subunit A; FAT1—Fatty Acid Translocase 1; Caspase-8—Caspase-8; CIS (deleted NKCs)—IFNɤ—Interferon Gamma; TNF—Tumor Necrosis Factor; ATG7—Autophagy-Related 7; PDPN—Podoplanin; IMP1—Insulin-Like Growth Factor 2 mRNA-Binding Protein 1; PIN1—Peptidyl-Prolyl Cis-Trans Isomerase NIMA-Interacting 1; ATM—Ataxia Telangiectasia Mutated; PTEN—Phosphatase and Tensin Homolog; p85α—Phosphoinositide-3-Kinase Regulatory Subunit Alpha; XIAP—X-Linked Inhibitor of Apoptosis; RGS4—Regulator of G Protein Signaling 4; MMP2—Matrix Metalloproteinase-2; GLI1—GLI Family Zinc Finger 1; and PI3K/Akt—Phosphoinositide 3-Kinase/Protein Kinase B.

**Table 2:** Overview of studies investigating CRISPR/Cas9 gene editing for the purpose of regulating the cell interphase in GBM malignant cells.

Reference Year	Targeted Gene	Targeted Molecules/Focus	Targeted Function	CRISPR-Cas9 Gene Editing	Therapy Efficiency/Outcome
Cell proliferation					
Fierro et al. [62] 2022	PD-L1	PD-1	proliferation, invasion, and macrophage polarization	Knockout	Dual-sgRNAs with repair template caused a 64% reduction in PD-L1 protein levels in U87 cells.
Lumibao et al. [63] 2023	CHCHD2	EGFRvIII	mitochondrial respiration, glutathione status, and cell growth inhibition	Knockout	CRISPR-Cas9 knockout of CHCHD2 in EGFRvIII-expressing U87 cells altered mitochondrial respiration, glutathione status, and decreased cell growth and invasion under normoxic and hypoxic conditions.
Toledano et al. [64] 2023	Plexin-A2	β-galactosidase, MAPK, FARP2	cytoskeletal organization, cell flattening, and cell cycle arrest	Knockout	Plexin-A2’s proproliferative effects are mediated via FARP2, FYN, and the GTPase activating (GAP) domain in its intracellular domain.
Gallo et al. [13] 2023	14-3-3β	Bad, FBI1, Raf-1, Cdc25b	proliferation and spheroid formation	Knockout	14-3-3β knockout resulted in impaired proliferation and decreased cells within a 3D-spheroid of U87MG cells.
Meng et al. [65] 2018	CDK7	n/d	growth	Knockout	
Guda et al. [59] 2020	RGS4	MMP2	proliferation	Knockout	
Zhang et al. [66] 2020	Nanos3	CD133, Oct4	proliferation, migration, and chemoresistance	Knockdown	Nanos3 deletion reduced proliferation, migration, and invasion of GBM cells in vitro (*p* < 0.05), increased sensitivity to DOX and TMZ (*p* < 0.05), and inhibited subcutaneous xenograft tumor growth in vivo (*p* < 0.001).
Godoy et al. [67] 2020	NRF2	SOD	self-renewal and cell proliferation	Knockdown	NRF2 knockdown resulted in less self-renewal, more differentiated cells, and decreased proliferation after irradiation with low- and high-dose rate gamma rays.
Zhang et al. [68] 2020	Dazl	CD133/Oct4/Nanog/Sox2 regulatory axis	proliferation	Knockout	Knocking down Dazl in A172, U251, and LN229 cell lines resulted in reduced proliferation rates and decreased migration of Dazl+/− cells compared to Dazl WT cells (*p* < 0.05) in both instances.
Liu et al. [69] 2018	ERβ	ERβ1, ERβ2, ERβ3, ERβ4, ERβ5 (exon 8), mTOR and STAT-3	proliferation and apoptosis	Knockout	ERβ KO cells exhibited high migratory and invasive potentials, while ERβ1 re-expression reduced this phenotype.
Cell renewal					
Bulstrode et al. [70]	Foxo3	FOXG1, SOX2, EGFR, EGFRvIII	differentiation	Knockdown	FOXG1 deletion in patient-derived GBM stem cells increased astrocyte differentiation and up-regulated FOXO3 in vivo.
Saent—Antonanzas [71] 2021	SRR2	SOX2	self-renowal capacity	Deletion	SOX2 ablation attenuated proliferation, and mutant cells could not be expanded in vitro. SRR2-deleted GBM cells displayed reduced SOX2 expression, decreased proliferative activity, and inhibited tumor initiation and growth in vivo.
Song et al. [72] 2019	SRSF3	SR proteins	glioma-associated alternative splicing	Knockout	ETV1 gene showed exon skipping at exon 7, and NDE1 gene showed replacement of terminal exon 9 with exon 9′, increasing their oncogenic activity in GSCs.
Cell migration					
Ogawa et al. [73] 2018	TP53	n/a	migration	Recombination	
Smolkin et al. [74] 2018	NRP2	Plexin-A4 Plexin-D1 Semaphorin-3C	migration	Knockout	Sema3D and Sema3G could not transduce signals without neuropilins.
Prolo et al. [75] 2019	MAP4K4	n/d	migration and invasion	Knockout	MAP4K4 knockout led to a 41% reduction in invasion compared to U138-Cas9 control.
Wang et al. [76] 2021	BRG1	STAT3	migration, proliferation, and TMZ resistance	Knockout	BRG1-KO inhibited GBM cell migration and invasion, sensitizing cells to TMZ.
Shao et al. [77] 2022	PIK3CD	PAK3 PLEK2	migration and invasion	Knockout	SD2 and SD13 cells did not form any noticeable xenograft tumor even 26 days after implantation, whereas xenograft tumors could be clearly observed 7 days after implantation in the U87-MG
Chen et al. [78] 2023	THBS1	TNF	proliferation and migration	Knockout	THBS1 gene knockout promoted proliferation and migration in U251 cells and GSCs.
Fierro et al. [62] 2022	PD-L1	PD-L1	proliferation, growth, invasion, and migration	Deletion	PD-L1 deletion reduced BrdU + proliferating U87 cells and prevented cell invasion.
Ozyerli-Gokna et al. [79] 2022	ASH2L	SET1/MLL	proliferation and migration	Knockout	ASH2L knockout resulted in significant gene expression changes.
Nieland et al. [80] 2022	miR21	SOX2	migration, invasion, and proliferation	Knockout	Proliferation significantly decreased in miR-21 KO in GL261, CT2A, and U87 cells. CT2A cells showed increased migration and invasion over GL261 cells.
Uceda-Castro et al. [81] 2022	GFAP	GFAPα, GFAPδ	invasion	Knockout	GFAPδ and GFAPα isoforms differentially regulate glioma cell dynamics. Depletion of either isoform increases migratory capacity, with distinct invasion patterns into brain tissue.

PD-L1—Programmed Death-Ligand 1; PD-1—Programmed Cell Death Protein 1; CHCHD2—Coiled-Coil-Helix-Coiled-Coil-Helix Domain Containing 2; EGFRvIII—Epidermal Growth Factor Receptor Variant III; Plexin-A2—A2 Isoform of the Plexin Family; β-galactosidase—Beta-Galactosidase; MAPK—Mitogen-Activated Protein Kinase; FARP2—FERM, RhoGEF (ARHGEF), and Pleckstrin Domain Protein 2; 14-3-3β—14-3-3 Protein Beta; Bad—Bcl-2-Associated Death Promoter; FBI1—Factor That Binds to Induce Interphase; Raf-1—Rapidly Accelerated Fibrosarcoma 1; Cdc25b—Cell Division Cycle 25 Homolog B; CDK7—Cyclin-Dependent Kinase 7; n/d—Not Defined; RGS4—Regulator of G Protein Signaling 4; MMP2—Matrix Metalloproteinase 2; Nanos3—Nanos Homolog 3; CD133—Cluster of Differentiation 133; Oct4—Octamer-Binding Transcription Factor 4; NRF2—Nuclear Factor Erythroid 2-Related Factor 2; SOD—Superoxide Dismutase; Dazl—Deleted in Azoospermia-Like; ERβ—Estrogen Receptor Beta; mTOR—Mechanistic Target of Rapamycin; STAT-3—Signal Transducer and Activator of Transcription 3; Cell renewal—Process of Renewing Cells; Foxo3—Forkhead Box O3; FOXG1—Forkhead Box G1; SOX2—SRY-Box Transcription Factor 2; EGFR—Epidermal Growth Factor Receptor; SRR2—Serine/Arginine Repetitive Matrix 2; SRSF3—Serine/Arginine-Rich Splicing Factor 3; SR proteins—Serine/Arginine-Rich Proteins; Cell migration—Process of Cell Movement; TP53—Tumor Protein P53; n/a—Not Applicable; NRP2—Neuropilin 2; Plexin-A4—A4 Isoform of the Plexin Family; Plexin-D1—D1 Isoform of the Plexin Family; Semaphorin-3C—Semaphorin 3C; MAP4K4—Mitogen-Activated Protein Kinase Kinase Kinase Kinase 4; BRG1—Brahma-Related Gene 1; STAT3—Signal Transducer and Activator of Transcription 3; PIK3CD—Phosphatidylinositol-4,5-Bisphosphate 3-Kinase Catalytic Subunit Delta; PAK3—p21-Activated Kinase 3; PLEK2—Pleckstrin 2; THBS1—Thrombospondin 1; TNF—Tumor Necrosis Factor; ASH2L—ASH2 (Absent, Small, or Homeotic)-Like; SET1/MLL—SET Domain Containing 1/Mixed Lineage Leukemia; miR21—microRNA 21; GFAP—Glial Fibrillary Acidic Protein; GFAPα—Alpha Isoform of Glial Fibrillary Acidic Protein; and GFAPδ—Delta Isoform of Glial Fibrillary Acidic Protein.

**Table 3:** Overview of studies investigating CRISPR/Cas9 gene editing for the purpose of regulating the microenvironment in GBM malignant cells.

Reference Year	Targeted Gene	Targeted Molecules	Targeted Function	CRISPR-Cas9 Gene Editing	Therapy Efficiency or Outcome
Angiogenesis					
Han et al. [82] 2017	Notch1	n/d	hypoxia, angiogenesis, and tumor growth	Knockdown	Xenografts with Notch1 downregulation reached 6 x the starting volume in 18.3 days, while control xenografts took 13.4 days.
Eisemann et al. [83] 2019	PDPN	PDPN	mediates the maturation and integrity of the developing vasculature in the murine brain in interaction with C-type lectin-like receptor 2 on platelets	Knockout	Similar rates of proliferation, apoptosis, angiogenesis, and invasion were observed in control and podoplanin-deleted tumors.
Szymura et al. [84] 2020	DDX39B	NF-κB	regulation of the extracellular ECM and promotes angiogenesis	Knockdown	CRISPR-mediated DDX39B depletion increased p65 phosphorylation, while MAVS knockdown reduced this phosphorylation; loss of DDX39B rendered U87 cells highly resistant to TMZ.
Lu et al. [85] 2019	BIG1, BIG2	VEGF	angiogenesis	Knockdown	BIG1 and BIG2 knockdown significantly decreased VEGF mRNA and protein levels in GBM U251 cells and HUVECs.
Lee et al. [86] 2023	ANGPT2	VEGFR2	normal-to-tumor vascular transition	Knockout	Treatment with the agonistic anti-Tie2 antibody, 4E2, resulted in vascular normalization throughout GBM tissues.
Inflammation					
Nakazawa et al. [55]	CIS	NKCs	Enhances NKCs effects	Knockout	The NK mock group showed longer survival compared to the NB group (mOS: 41.0 days vs. 56.5 days). The NK dCIS group exhibited prolonged OS compared to the NK mock group (mOS: 79.5 days).
Wei et al. [87]	OPN	M2 macrophages	M2 macrophages reduction and T-lymphocite effector activity elevation	Knockout	OPN deficiency in innate immune or glioma cells reduced M2 macrophages and elevated T cell effector activity infiltrating the glioma.
Chen et al. [88]	AIM2	IL-1β, IL-18	Pyroptosis (infammatory programmed cell death)	Knockdown	AIM2 immunoreactivity concentrated in the tumor core in the absence of PCNA immunodetection, showing a predominant 52 kDa immunoreactive band on western blot.

Notch1—Notch Receptor 1; n/d—Not Defined; PDPN—Podoplanin; DDX39B—DEAD-Box Helicase 39B; NF-κB—Nuclear Factor Kappa B; BIG1 and BIG2—Brefeldin A-Inhibited Guanine Nucleotide-Exchange Proteins 1 and 2; VEGF—Vascular Endothelial Growth Factor; ANGPT2—Angiopoietin-2; VEGFR2—Vascular Endothelial Growth Factor Receptor 2; Inflammation—Process of Inflammatory Response; CIS—Cytokine-Inducible SH2-Containing Protein; NKCs—Natural Killer Cells; OPN—Osteopontin; M2 (macrophages)—M2 Phenotype of Macrophages; AIM2—Absent in Melanoma 2; IL-1β—Interleukin-1 Beta; and IL-18—Interleukin-18.

**Table 4:** Contribution of CRISPR/Cas9 technology in alleviating therapy resistance of GBM.

Reference Year	Targeted Gene	Targeted Molecules/Focus	Targeted Function	CRISPR-Cas9 Gene Editing	Therapy Efficiency or Outcome
Wu et al. [89] 2020	ALDH1A3	ALDHs	TMZ resistance	Knockdown	The observed difference was particularly significant at dosages ≤ 300 μM.
Han et al. [90] 2023	MGMT	MGMT	TMZ resistance	Knockdown	T98G and LN18 cells displayed a dose-dependent decrease in viability, with IC50 values of 475.6 µM and 424.7 µM, respectively.
Tong et al. [91] 2023	MUC1	EGFRvIII	TMZ resistance	Knockdown	EGFRvIII was localized in the nucleus after TMZ treatment, consistent with its reported role in assisting DNA damage repair during chemotherapy and radiation.
Liu et al. [92] 2023	GSS	Angiopep-2	Radiotherapy resistance	Knockout	GSS perturbation in glioma cells demonstrated significant antitumor activity when combined with radiotherapy.
Rocha et al. [93] 2020	MSH2, PTCH2, CLCA2, FZD6, CTNNB1, NRF2	Transmembrane proteins	TMZ resistance	Knockout	Silencing the top three genes (MSH2, PTCH2, and CLCA2) confirmed cell protection from TMZ-induced death.
Yin et al. [94]	HPRT1	AMPK	TMZ resistance	Knockout	Combining HPRT1 depletion with TMZ treatment achieved the longest survival extension.

ALDH1A3—Aldehyde Dehydrogenase 1A3; ALDHs—Aldehyde Dehydrogenases; MGMT—O-6-Methylguanine-DNA Methyltransferase; MUC1—Mucin 1; EGFRvIII—Epidermal Growth Factor Receptor Variant III; GSS—Glutathione Synthetase; Angiopep-2—Angiopep-2 Peptide; MSH2—MutS Homolog 2; PTCH2—Patched 2; CLCA2—Calcium-Activated Chloride Channel Family Member 2; FZD6—Frizzled Class Receptor 6; CTNNB1—Catenin Beta 1; NRF2—Nuclear Factor Erythroid 2-Related Factor 2; Transmembrane proteins—Proteins with Transmembrane Domains; HPRT1—Hypoxanthine Phosphoribosyltransferase 1; and AMPK—AMP-Activated Protein Kinase.

## References

[1] Begagić E., Pugonja R., Bečulić H., Čeliković A., Tandir Lihić L., Kadić Vukas S., Čejvan L., Skomorac R., Selimović E., Jaganjac B. (2023). Molecular Targeted Therapies in Glioblastoma Multiforme: A Systematic Overview of Global Trends and Findings. Brain Sci..

[2] Stoyanov G.S., Lyutfi E., Georgieva R., Georgiev R., Dzhenkov D.L., Petkova L., Ivanov B.D., Kaprelyan A., Ghenev P. (2022). Reclassification of Glioblastoma Multiforme According to the 2021 World Health Organization Classification of Central Nervous System Tumors: A Single Institution Report and Practical Significance. Cureus.

[3] Jain K.K. (2018). A Critical Overview of Targeted Therapies for Glioblastoma. Front. Oncol..

[4] Tatebayashi K., Nakayama N., Sakamoto D., Iida T., Ono S., Matsuda I., Enomoto Y., Tanaka M., Fujita M., Hirota S. (2023). Clinical Significance of Early Venous Filling Detected via Preoperative Angiography in Glioblastoma. Cancers.

[5] Angom R.S., Nakka N.M.R., Bhattacharya S. (2023). Advances in Glioblastoma Therapy: An Update on Current Approaches. Brain Sci..

[6] Agosti E., Zeppieri M., De Maria L., Tedeschi C., Fontanella M.M., Panciani P.P., Ius T. (2023). Glioblastoma Immunotherapy: A Systematic Review of the Present Strategies and Prospects for Advancements. Int. J. Mol. Sci..

[7] Riemenschneider M.J., Jeuken J.W., Wesseling P., Reifenberger G. (2010). Molecular diagnostics of gliomas: State of the art. Acta Neuropathol..

[8] Isachesku E., Braicu C., Pirlog R., Kocijancic A., Busuioc C., Pruteanu L.-L., Pandey D.P., Berindan-Neagoe I. (2023). The Role of Non-Coding RNAs in Epigenetic Dysregulation in Glioblastoma Development. Int. J. Mol. Sci..

[9] Senhaji N., Squalli Houssaini A., Lamrabet S., Louati S., Bennis S. (2022). Molecular and Circulating Biomarkers in Patients with Glioblastoma. Int. J. Mol. Sci..

[10] Khlidj Y. (2023). What did CRISPR-Cas9 accomplish in its first 10 years?. Biochem. Med..

[11] Peixoto J., Príncipe C., Pestana A., Osório H., Pinto M.T., Prazeres H., Soares P., Lima R.T. (2023). Using a Dual CRISPR/Cas9 Approach to Gain Insight into the Role of LRP1B in Glioblastoma. Int. J. Mol. Sci..

[12] Brennan C.W., Verhaak R.G., McKenna A., Campos B., Noushmehr H., Salama S.R., Zheng S., Chakravarty D., Sanborn J.Z., Berman S.H. (2013). The somatic genomic landscape of glioblastoma. Cell.

[13] Gallo K., Srinageshwar B., Ward A., Diola C., Dunbar G., Rossignol J., Bakke J. (2023). Inducible Knockout of 14-3-3β Attenuates Proliferation and Spheroid Formation in a Human Glioblastoma Cell Line U87MG. Brain Sci..

[14] Motoche-Monar C., Ordoñez J.E., Chang O., Gonzales-Zubiate F.A. (2023). gRNA Design: How Its Evolution Impacted on CRISPR/Cas9 Systems Refinement. Biomolecules.

[15] Ding S., Liu J., Han X., Tang M. (2023). CRISPR/Cas9-Mediated Genome Editing in Cancer Therapy. Int. J. Mol. Sci..

[16] Begagić E., Pugonja R., Bečulić H., Selimović E., Skomorac R., Saß B., Pojskić M. (2023). The new era of spinal surgery: Exploring the utilization of exoscopes as a viable alternative to operative microscopes—A systematic review and meta-analysis. World Neurosurg..

[17] Begagić E., Bečulić H., Skomorac R., Pojskić M. (2023). Accessible Spinal Surgery: Transformation Through the Implementation of Exoscopes As Substitutes for Conventional Microsurgery in Low- and Middle-Income Settings. Cureus.

[18] Li C., Brant E., Budak H., Zhang B. (2021). CRISPR/Cas: A Nobel Prize award-winning precise genome editing technology for gene therapy and crop improvement. J. Zhejiang Univ. Sci. B.

[19] Mohammadzadeh I., Qujeq D., Yousefi T., Ferns G.A., Maniati M., Vaghari-Tabari M. (2020). CRISPR/Cas9 gene editing: A new therapeutic approach in the treatment of infection and autoimmunity. IUBMB Life.

[20] Gostimskaya I. (2022). CRISPR-Cas9: A History of Its Discovery and Ethical Considerations of Its Use in Genome Editing. Biochemistry.

[21] Ishino Y., Krupovic M., Forterre P. (2018). History of CRISPR-Cas from Encounter with a Mysterious Repeated Sequence to Genome Editing Technology. J. Bacteriol..

[22] Kozovska Z., Rajcaniova S., Munteanu P., Dzacovska S., Demkova L. (2021). CRISPR: History and perspectives to the future. Biomed. Pharmacother..

[23] Janik E., Niemcewicz M., Ceremuga M., Krzowski L., Saluk-Bijak J., Bijak M. (2020). Various Aspects of a Gene Editing System—CRISPR–Cas9. Int. J. Mol. Sci..

[24] Xing H., Meng L.H. (2020). CRISPR-cas9: A powerful tool towards precision medicine in cancer treatment. Acta Pharmacol. Sin..

[25] Jiang C., Meng L., Yang B., Luo X. (2020). Application of CRISPR/Cas9 gene editing technique in the study of cancer treatment. Clin. Genet..

[26] Wang S.-W., Gao C., Zheng Y.-M., Yi L., Lu J.-C., Huang X.-Y., Cai J.-B., Zhang P.-F., Cui Y.-H., Ke A.-W. (2022). Current applications and future perspective of CRISPR/Cas9 gene editing in cancer. Mol. Cancer.

[27] Hazafa A., Mumtaz M., Farooq M.F., Bilal S., Chaudhry S.N., Firdous M., Naeem H., Ullah M.O., Yameen M., Mukhtiar M.S. (2020). CRISPR/Cas9: A powerful genome editing technique for the treatment of cancer cells with present challenges and future directions. Life Sci..

[28] Hsu M.N., Chang Y.H., Truong V.A., Lai P.L., Nguyen T.K.N., Hu Y.C. (2019). CRISPR technologies for stem cell engineering and regenerative medicine. Biotechnol. Adv..

[29] Jhu M.-Y., Ellison E.E., Sinha N.R. (2023). CRISPR gene editing to improve crop resistance to parasitic plants. Front. Genome Ed..

[30] Hirsch F., Iphofen R., Koporc Z. (2019). Ethics assessment in research proposals adopting CRISPR technology. Biochem. Med..

[31] Gumer J.M. (2019). The Wisdom of Germline Editing: An Ethical Analysis of the Use of CRISPR-Cas9 to Edit Human Embryos. New. Bioeth..

[32] Sauvagère S., Siatka C. (2023). CRISPR-Cas: &lsquo;The Multipurpose Molecular Tool&rsquo; for Gene Therapy and Diagnosis. Genes.

[33] Gupta S., Kumar A., Patel R., Kumar V. (2021). Genetically modified crop regulations: Scope and opportunity using the CRISPR-Cas9 genome editing approach. Mol. Biol. Rep..

[34] Zhang B. (2021). CRISPR/Cas gene therapy. J. Cell. Physiol..

[35] Yu W., Wu Z. (2019). Use of AAV Vectors for CRISPR-Mediated In Vivo Genome Editing in the Retina. Methods Mol. Biol..

[36] Asmamaw Mengstie M. (2022). Viral Vectors for the in Vivo Delivery of CRISPR Components: Advances and Challenges. Front. Bioeng. Biotechnol..

[37] Wang L., Zheng W., Liu S., Li B., Jiang X. (2019). Delivery of CRISPR/Cas9 by Novel Strategies for Gene Therapy. Chembiochem.

[38] Uddin F., Rudin C.M., Sen T. (2020). CRISPR Gene Therapy: Applications, Limitations, and Implications for the Future. Front. Oncol..

[39] Lim J.M., Kim H.H. (2022). Basic Principles and Clinical Applications of CRISPR-Based Genome Editing. Yonsei Med. J..

[40] Horodecka K., Düchler M. (2021). CRISPR/Cas9: Principle, Applications, and Delivery through Extracellular Vesicles. Int. J. Mol. Sci..

[41] Tabassum T., Pietrogrande G., Healy M., Wolvetang E.J. (2023). CRISPR-Cas9 Direct Fusions for Improved Genome Editing via Enhanced Homologous Recombination. Int. J. Mol. Sci..

[42] Yip B.H. (2020). Recent Advances in CRISPR/Cas9 Delivery Strategies. Biomolecules.

[43] Reuven N., Adler J., Myers N., Shaul Y. (2021). CRISPR Co-Editing Strategy for Scarless Homology-Directed Genome Editing. Int. J. Mol. Sci..

[44] Loureiro A., da Silva G.J. (2019). CRISPR-Cas: Converting A Bacterial Defence Mechanism into A State-of-the-Art Genetic Manipulation Tool. Antibiotics.

[45] Karginov A.V., Tarutina M.G., Lapteva A.R., Pakhomova M.D., Galliamov A.A., Filkin S.Y., Fedorov A.N., Agaphonov M.O. (2023). A Split-Marker System for CRISPR-Cas9 Genome Editing in Methylotrophic Yeasts. Int. J. Mol. Sci..

[46] Kang X., Wang Y., Liu P., Huang B., Zhou B., Lu S., Geng W., Tang H. (2023). Progresses, Challenges, and Prospects of CRISPR/Cas9 Gene-Editing in Glioma Studies. Cancers.

[47] Qazi M.A., Vora P., Venugopal C., Sidhu S.S., Moffat J., Swanton C., Singh S.K. (2017). Intratumoral heterogeneity: Pathways to treatment resistance and relapse in human glioblastoma. Ann. Oncol..

[48] Lake J.A., Donson A.M., Prince E., Davies K.D., Nellan A., Green A.L., Mulcahy Levy J., Dorris K., Vibhakar R., Hankinson T.C. (2020). Targeted fusion analysis can aid in the classification and treatment of pediatric glioma, ependymoma, and glioneuronal tumors. Pediatr. Blood Cancer.

[49] Wang X., Wang X., Li J., Liang J., Ren X., Yun D., Liu J., Fan J., Zhang Y., Zhang J. (2023). PDPN contributes to constructing immunosuppressive microenvironment in IDH wildtype glioma. Cancer Gene Therapy.

[50] Nayak S., Aich M., Kumar A., Sengupta S., Bajad P., Dhapola P., Paul D., Narta K., Purkrait S., Mehani B. (2018). Novel internal regulators and candidate miRNAs within miR-379/miR-656 miRNA cluster can alter cellular phenotype of human glioblastoma. Sci. Rep..

[51] Rodvold J.J., Xian S., Nussbacher J., Tsui B., Cameron Waller T., Searles S.C., Lew A., Jiang P., Babic I., Nomura N. (2020). IRE1α and IGF signaling predict resistance to an endoplasmic reticulum stress-inducing drug in glioblastoma cells. Sci. Rep..

[52] Vu H.T., Kobayashi M., Hegazy A.M., Tadokoro Y., Ueno M., Kasahara A., Takase Y., Nomura N., Peng H., Ito C. (2018). Autophagy inhibition synergizes with calcium mobilization to achieve efficient therapy of malignant gliomas. Cancer Sci..

[53] Peng H., Du B., Jiang H., Gao J. (2016). Over-expression of CHAF1A promotes cell proliferation and apoptosis resistance in glioblastoma cells via AKT/FOXO3a/Bim pathway. Biochem. Biophys. Res. Commun..

[54] Kranz D., Boutros M. (2014). A synthetic lethal screen identifies FAT1 as an antagonist of caspase-8 in extrinsic apoptosis. Embo J..

[55] Nakazawa T., Morimoto T., Maeoka R., Matsuda R., Nakamura M., Nishimura F., Ouji N., Yamada S., Nakagawa I., Park Y.S. (2023). CIS deletion by CRISPR/Cas9 enhances human primary natural killer cell functions against allogeneic glioblastoma. J. Exp. Clin. Cancer Res..

[56] Zielke S., Meyer N., Mari M., Abou-El-Ardat K., Reggiori F., van Wijk S.J.L., Kögel D., Fulda S. (2018). Loperamide, pimozide, and STF-62247 trigger autophagy-dependent cell death in glioblastoma cells. Cell. Death Dis..

[57] Maggio J., Cardama G.A., Armando R.G., Balcone L., Sobol N.T., Gomez D.E., Mengual Gómez D.L. (2023). Key role of PIN1 in telomere maintenance and oncogenic behavior in a human glioblastoma model. Oncol. Rep..

[58] Ali R., Alabdullah M., Miligy I., Normatova M., Babaei-Jadidi R., Nateri A.S., Rakha E.A., Madhusudan S. (2019). ATM Regulated PTEN Degradation Is XIAP E3 Ubiquitin Ligase Mediated in p85α Deficient Cancer Cells and Influence Platinum Sensitivity. Cells.

[59] Guda M.R., Velpula K.K., Asuthkar S., Cain C.P., Tsung A.J. (2020). Targeting RGS4 Ablates Glioblastoma Proliferation. Int. J. Mol. Sci..

[60] Ranjan A., Srivastava S.K. (2017). Penfluridol suppresses glioblastoma tumor growth by Akt-mediated inhibition of GLI1. Oncotarget.

[61] Esemen Y., Awan M., Parwez R., Baig A., Rahman S., Masala I., Franchini S., Giakoumettis D. (2022). Molecular Pathogenesis of Glioblastoma in Adults and Future Perspectives: A Systematic Review. Int. J. Mol. Sci..

[62] Fierro J., DiPasquale J., Perez J., Chin B., Chokpapone Y., Tran A.M., Holden A., Factoriza C., Sivagnanakumar N., Aguilar R. (2022). Dual-sgRNA CRISPR/Cas9 knockout of PD-L1 in human U87 glioblastoma tumor cells inhibits proliferation, invasion, and tumor-associated macrophage polarization. Sci. Rep..

[63] Lumibao J.C., Haak P.L., Kolossov V.L., Chen J.E., Stutchman J., Ruiz A., Sivaguru M., Sarkaria J.N., Harley B.A.C., Steelman A.J. (2023). CHCHD2 mediates glioblastoma cell proliferation, mitochondrial metabolism, hypoxia-induced invasion and therapeutic resistance. Int. J. Oncol..

[64] Toledano S., Sabag A.D., Ilan N., Liburkin-Dan T., Kessler O., Neufeld G. (2023). Plexin-A2 enables the proliferation and the development of tumors from glioblastoma derived cells. Cell Death Dis..

[65] Harutyunyan A.S., Krug B., Chen H., Papillon-Cavanagh S., Zeinieh M., De Jay N., Deshmukh S., Chen C.C.L., Belle J., Mikael L.G. (2019). H3K27M induces defective chromatin spread of PRC2-mediated repressive H3K27me2/me3 and is essential for glioma tumorigenesis. Nat. Commun..

[66] Zhang F., Liu R., Liu C., Zhang H., Lu Y. (2020). Nanos3, a cancer-germline gene, promotes cell proliferation, migration, chemoresistance, and invasion of human glioblastoma. Cancer Cell. Int..

[67] Godoy P.R.D.V., Pour Khavari A., Rizzo M., Sakamoto-Hojo E.T., Haghdoost S. (2020). Targeting NRF2, Regulator of Antioxidant System, to Sensitize Glioblastoma Neurosphere Cells to Radiation-Induced Oxidative Stress. Oxidative Med. Cell. Longev..

[68] Zhang F., Liu R., Zhang H., Liu C., Liu C., Lu Y. (2020). Suppressing Dazl modulates tumorigenicity and stemness in human glioblastoma cells. BMC Cancer.

[69] Liu J., Sareddy G.R., Zhou M., Viswanadhapalli S., Li X., Lai Z., Tekmal R.R., Brenner A., Vadlamudi R.K. (2018). Differential Effects of Estrogen Receptor β Isoforms on Glioblastoma Progression. Cancer Res..

[70] Bulstrode H., Johnstone E., Marques-Torrejon M.A., Ferguson K.M., Bressan R.B., Blin C., Grant V., Gogolok S., Gangoso E., Gagrica S. (2017). Elevated FOXG1 and SOX2 in glioblastoma enforces neural stem cell identity through transcriptional control of cell cycle and epigenetic regulators. Genes. Dev..

[71] Saenz-Antoñanzas A., Moncho-Amor V., Auzmendi-Iriarte J., Elua-Pinin A., Rizzoti K., Lovell-Badge R., Matheu A. (2021). CRISPR/Cas9 Deletion of SOX2 Regulatory Region 2 (SRR2) Decreases SOX2 Malignant Activity in Glioblastoma. Cancers.

[72] Song X., Wan X., Huang T., Zeng C., Sastry N., Wu B., James C.D., Horbinski C., Nakano I., Zhang W. (2019). SRSF3-Regulated RNA Alternative Splicing Promotes Glioblastoma Tumorigenicity by Affecting Multiple Cellular Processes. Cancer Res..

[73] Ogawa J., Pao G.M., Shokhirev M.N., Verma I.M. (2018). Glioblastoma Model Using Human Cerebral Organoids. Cell. Rep..

[74] Smolkin T., Nir-Zvi I., Duvshani N., Mumblat Y., Kessler O., Neufeld G. (2018). Complexes of plexin-A4 and plexin-D1 convey semaphorin-3C signals to induce cytoskeletal collapse in the absence of neuropilins. J. Cell. Sci..

[75] Prolo L.M., Li A., Owen S.F., Parker J.J., Foshay K., Nitta R.T., Morgens D.W., Bolin S., Wilson C.M., Vega L.J. (2019). Targeted genomic CRISPR-Cas9 screen identifies MAP4K4 as essential for glioblastoma invasion. Sci. Rep..

[76] Wang Y., Yang C.H., Schultz A.P., Sims M.M., Miller D.D., Pfeffer L.M. (2021). Brahma-Related Gene-1 (BRG1) promotes the malignant phenotype of glioblastoma cells. J. Cell. Mol. Med..

[77] Shao W., Azam Z., Guo J., To S.S.T. (2022). Oncogenic potential of PIK3CD in glioblastoma is exerted through cytoskeletal proteins PAK3 and PLEK2. Lab. Investig..

[78] Chen L., Fang W., Chen W., Wei Y., Ding J., Li J., Lin J., Wu Q. (2023). Deciphering the molecular mechanism of the THBS1 gene in the TNF signaling axis in glioma stem cells. Cell. Signal..

[79] Ezgi O.-G., Ezgi Yagmur K., Ali Cenk A., Ipek B., Ahmet C., Sheikh N., Martin B., Fidan S.-P., Tunc M., Can A. (2022). Epigenetic-focused CRISPR/Cas9 screen identifies ASH2L as a regulator of glioblastoma cell survival. bioRxiv.

[80] Nieland L., van Solinge T.S., Cheah P.S., Morsett L.M., El Khoury J., Rissman J.I., Kleinstiver B.P., Broekman M.L.D., Breakefield X.O., Abels E.R. (2022). CRISPR-Cas knockout of miR21 reduces glioma growth. Mol. Ther. Oncolytics.

[81] Uceda-Castro R., van Asperen J.V., Vennin C., Sluijs J.A., van Bodegraven E.J., Margarido A.S., Robe P.A.J., van Rheenen J., Hol E.M. (2022). GFAP splice variants fine-tune glioma cell invasion and tumour dynamics by modulating migration persistence. Sci. Rep..

[82] Han N., Hu G., Shi L., Long G., Yang L., Xi Q., Guo Q., Wang J., Dong Z., Zhang M. (2017). Notch1 ablation radiosensitizes glioblastoma cells. Oncotarget.

[83] Eisemann T., Costa B., Harter P.N., Wick W., Mittelbronn M., Angel P., Peterziel H. (2019). Podoplanin expression is a prognostic biomarker but may be dispensable for the malignancy of glioblastoma. Neuro-Oncol..

[84] Szymura S.J., Bernal G.M., Wu L., Zhang Z., Crawley C.D., Voce D.J., Campbell P.A., Ranoa D.E., Weichselbaum R.R., Yamini B. (2020). DDX39B interacts with the pattern recognition receptor pathway to inhibit NF-κB and sensitize to alkylating chemotherapy. BMC Biol..

[85] Lu F.I., Wang Y.T., Wang Y.S., Wu C.Y., Li C.C. (2019). Involvement of BIG1 and BIG2 in regulating VEGF expression and angiogenesis. FASEB J..

[86] Lee E., Lee E.A., Kong E., Chon H., Llaiqui-Condori M., Park C.H., Park B.Y., Kang N.R., Yoo J.S., Lee H.S. (2023). An agonistic anti-Tie2 antibody suppresses the normal-to-tumor vascular transition in the glioblastoma invasion zone. Exp. Mol. Med..

[87] Wei J., Marisetty A., Schrand B., Gabrusiewicz K., Hashimoto Y., Ott M., Grami Z., Kong L.Y., Ling X., Caruso H. (2019). Osteopontin mediates glioblastoma-associated macrophage infiltration and is a potential therapeutic target. J. Clin. Investig..

[88] Chen P.A., Shrivastava G., Balcom E.F., McKenzie B.A., Fernandes J., Branton W.G., Wheatley B.M., Petruk K., van Landeghem F.K.H., Power C. (2019). Absent in melanoma 2 regulates tumor cell proliferation in glioblastoma multiforme. J. Neurooncol..

[89] Wu W., Wu Y., Mayer K., von Rosenstiel C., Schecker J., Baur S., Würstle S., Liesche-Starnecker F., Gempt J., Schlegel J. (2020). Lipid Peroxidation Plays an Important Role in Chemotherapeutic Effects of Temozolomide and the Development of Therapy Resistance in Human Glioblastoma. Transl. Oncol..

[90] Han X., Abdallah M.O.E., Breuer P., Stahl F., Bakhit Y., Potthoff A.L., Pregler B.E.F., Schneider M., Waha A., Wüllner U. (2023). Downregulation of MGMT expression by targeted editing of DNA methylation enhances temozolomide sensitivity in glioblastoma. Neoplasia.

[91] Tong F., Zhao J.X., Fang Z.Y., Cui X.T., Su D.Y., Liu X., Zhou J.H., Wang G.X., Qiu Z.J., Liu S.Z. (2023). MUC1 promotes glioblastoma progression and TMZ resistance by stabilizing EGFRvIII. Pharmacol. Res..

[92] Liu X., Cao Z., Wang W., Zou C., Wang Y., Pan L., Jia B., Zhang K., Zhang W., Li W. (2023). Engineered Extracellular Vesicle-Delivered CRISPR/Cas9 for Radiotherapy Sensitization of Glioblastoma. ACS Nano.

[93] Rocha C.R.R., Rocha A.R., Silva M.M., Gomes L.R., Latancia M.T., Andrade-Tomaz M., de Souza I., Monteiro L.K.S., Menck C.F.M. (2020). Revealing Temozolomide Resistance Mechanisms via Genome-Wide CRISPR Libraries. Cells.

[94] Yin J., Wang X., Ge X., Ding F., Shi Z., Ge Z., Huang G., Zhao N., Chen D., Zhang J. (2023). Hypoxanthine phosphoribosyl transferase 1 metabolizes temozolomide to activate AMPK for driving chemoresistance of glioblastomas. Nat. Commun..

[95] Álvarez M.M., Biayna J., Supek F. (2022). TP53-dependent toxicity of CRISPR/Cas9 cuts is differential across genomic loci and can confound genetic screening. Nat. Commun..

[96] Ihry R.J., Worringer K.A., Salick M.R., Frias E., Ho D., Theriault K., Kommineni S., Chen J., Sondey M., Ye C. (2018). p53 inhibits CRISPR-Cas9 engineering in human pluripotent stem cells. Nat. Med..

[97] Sinha S., Barbosa K., Cheng K., Leiserson M.D.M., Jain P., Deshpande A., Wilson D.M., Ryan B.M., Luo J., Ronai Z.e.A. (2021). A systematic genome-wide mapping of oncogenic mutation selection during CRISPR-Cas9 genome editing. Nat. Commun..

[98] Enache O.M., Rendo V., Abdusamad M., Lam D., Davison D., Pal S., Currimjee N., Hess J., Pantel S., Nag A. (2020). Cas9 activates the p53 pathway and selects for p53-inactivating mutations. Nat. Genet..

[99] Hwang W.Y., Fu Y., Reyon D., Maeder M.L., Tsai S.Q., Sander J.D., Peterson R.T., Yeh J.R., Joung J.K. (2013). Efficient genome editing in zebrafish using a CRISPR-Cas system. Nat. Biotechnol..

[100] Bečulić H., Begagić E., Skomorac R., Mašović A., Selimović E., Pojskić M. (2024). ChatGPT’s contributions to the evolution of neurosurgical practice and education: A systematic review of benefits, concerns and limitations. Med. Glas..

[101] Ayanoğlu F.B., Elçin A.E., Elçin Y.M. (2020). Bioethical issues in genome editing by CRISPR-Cas9 technology. Turk. J. Biol..

[102] Zuo E., Sun Y., Yuan T., He B., Zhou C., Ying W., Liu J., Wei W., Zeng R., Li Y. (2020). A rationally engineered cytosine base editor retains high on-target activity while reducing both DNA and RNA off-target effects. Nat. Methods.

[103] Yunta E. (2016). Ethical Issues in Genome Editing using Crispr/Cas9 System. J. Clin. Res. Bioeth..

[104] Ray U., Raghavan S.C. (2020). Modulation of DNA double-strand break repair as a strategy to improve precise genome editing. Oncogene.

[105] Stoltz K., Sinyuk M., Hale J.S., Wu Q., Otvos B., Walker K., Vasanji A., Rich J.N., Hjelmeland A.B., Lathia J.D. (2015). Development of a Sox2 reporter system modeling cellular heterogeneity in glioma. Neuro-Oncol..

[106] Xu P.F., Li C., Xi S.Y., Chen F.R., Wang J., Zhang Z.Q., Liu Y., Li X., Chen Z.P. (2022). Whole exome sequencing reveals the genetic heterogeneity and evolutionary history of primary gliomas and matched recurrences. Comput. Struct. Biotechnol. J..

[107] Liu C., Sage J.C., Miller M.R., Verhaak R.G., Hippenmeyer S., Vogel H., Foreman O., Bronson R.T., Nishiyama A., Luo L. (2011). Mosaic analysis with double markers reveals tumor cell of origin in glioma. Cell.

[108] Bhat K.P.L., Balasubramaniyan V., Vaillant B., Ezhilarasan R., Hummelink K., Hollingsworth F., Wani K., Heathcock L., James J.D., Goodman L.D. (2013). Mesenchymal differentiation mediated by NF-κB promotes radiation resistance in glioblastoma. Cancer Cell.

[109] Ene C.I., Holland E.C. (2015). Personalized medicine for gliomas. Surg. Neurol. Int..

[110] Rosenblum D., Gutkin A., Kedmi R., Ramishetti S., Veiga N., Jacobi A.M., Schubert M.S., Friedmann-Morvinski D., Cohen Z.R., Behlke M.A. (2020). CRISPR-Cas9 genome editing using targeted lipid nanoparticles for cancer therapy. Sci. Adv..

[111] Luo Y.L., Xu C.F., Li H.J., Cao Z.T., Liu J., Wang J.L., Du X.J., Yang X.Z., Gu Z., Wang J. (2018). Macrophage-Specific in Vivo Gene Editing Using Cationic Lipid-Assisted Polymeric Nanoparticles. ACS Nano.

[112] Tsuchida C.A., Brandes N., Bueno R., Trinidad M., Mazumder T., Yu B., Hwang B., Chang C., Liu J., Sun Y. (2023). Mitigation of chromosome loss in clinical CRISPR-Cas9-engineered T cells. Cell.

[113] Mehta A., Merkel O.M. (2020). Immunogenicity of Cas9 Protein. J. Pharm. Sci..

[114] Crudele J.M., Chamberlain J.S. (2018). Cas9 immunity creates challenges for CRISPR gene editing therapies. Nat. Commun..

[115] Yang S., Chang R., Yang H., Zhao T., Hong Y., Kong H.E., Sun X., Qin Z., Jin P., Li S. (2017). CRISPR/Cas9-mediated gene editing ameliorates neurotoxicity in mouse model of Huntington’s disease. J. Clin. Investig..

[116] Cribbs A.P., Perera S.M.W. (2017). Science and Bioethics of CRISPR-Cas9 Gene Editing: An Analysis Towards Separating Facts and Fiction. Yale J. Biol. Med..

[117] Tao J., Bauer D.E., Chiarle R. (2023). Assessing and advancing the safety of CRISPR-Cas tools: From DNA to RNA editing. Nat. Commun..

[118] Zha M.J., Cai C.E., He P.M. (2023). Outlook on the Security and Potential Improvements of CRISPR-Cas9. Mol. Biotechnol..

[119] Lu Y., Xue J., Deng T., Zhou X., Yu K., Deng L., Huang M., Yi X., Liang M., Wang Y. (2020). Safety and feasibility of CRISPR-edited T cells in patients with refractory non-small-cell lung cancer. Nat. Med..

[120] Deng H.X., Zhai H., Shi Y., Liu G., Lowry J., Liu B., Ryan É.B., Yan J., Yang Y., Zhang N. (2021). Efficacy and long-term safety of CRISPR/Cas9 genome editing in the SOD1-linked mouse models of ALS. Commun. Biol..

[121] Sledzinski P., Dabrowska M., Nowaczyk M., Olejniczak M. (2021). Paving the way towards precise and safe CRISPR genome editing. Biotechnol. Adv..

